# Differences in Electrodermal Activity in Depressed Bipolar Patients with High and Low Anxiety Levels

**DOI:** 10.1192/j.eurpsy.2025.441

**Published:** 2025-08-26

**Authors:** C. Valenzuela-Pascual, D. Hidalgo-Mazzei, A. Mas, G. Anmella, F. Corponi, E. Vieta

**Affiliations:** 1 Department of Psychiatry and Psychology, Hospital Clínic de Barcelona; 2 Bipolar and Depressive Disorders Unit, Institut d’Investigacions Biomèdiques August Pi I Sunyer (IDIBAPS); 3 Department of Medicine, School of Medicine and Health Sciences, Institute of Neurosciences (UBNeuro), University of Barcelona (UB), Barcelona, Spain; 4 School of Informatics, University of Edinburgh, Edinburgh, United Kingdom

## Abstract

**Introduction:**

Electrodermal activity (EDA) measures the skin’s electrical properties. It varies according to the sweat gland’s activity which responds to the sympathetic nervous system (Boucsein W. Electrodermal Activity. Berlin: Plenum Press; 2012). Previous literature has reported lower EDA during bipolar and unipolar depressive episodes (Sarchiapone M, et al. BMC Psychiatry 2018; 18: 22 & Valenzuela-Pascual C, et al. Acta Psychiatr Scand 2024). Historically, heightened anxiety has been correlated with increased EDA, although findings in this area have been inconsistent (Naveteur J, et al. Int J Psychophysiol 2005; 56(3): 261–269).

**Objectives:**

This study aimed to determine whether significant differences in EDA exist among depressed patients based on their levels of anxiety.

**Methods:**

We analysed EDA recordings from an E4 wearable device utilised by 29 depressed patients with bipolar disorder. They wore the device for a period of 48 hours without altering their daily routines. The tonic mean and phasic peaks parameters of EDA were extracted and analysed via a mixed-effects model for repeated measures, incorporating sleep state and anxiety level as variables. Anxiety levels were assessed based on the scores from item 10 on the Hamilton Rating Scale for depression, which reflects psychic anxiety.

**Results:**

The results indicated that anxiety levels did not significantly influence any of the models examined. However, in the phasic peaks model, there is a noteworthy interaction between anxiety level and sleep status (p < 0.01). Both models demonstrated a tendency towards increased EDA values in the high anxiety group although these findings did not reach statistical significance. This trend was consistent across both sleep states for the tonic mean model (Image 1). In contrast, the high anxiety group exhibited higher phasic peaks values (M [SD] = 2.33 [0.80-3.86]) compared to the low anxiety group (M [SD] = 2.33 [0.80-3.86]) only during wakefulness, although this difference was not statistically significant (p > 0.05) (Image 2).

**Image 1:**

**Figure:**
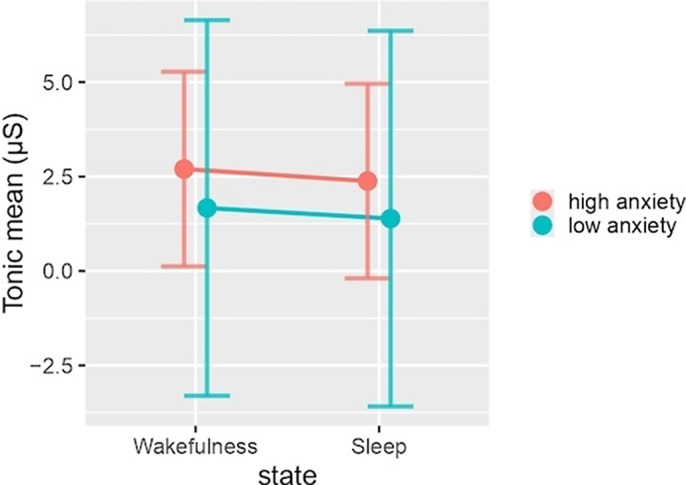

**Image 2:**

**Figure:**
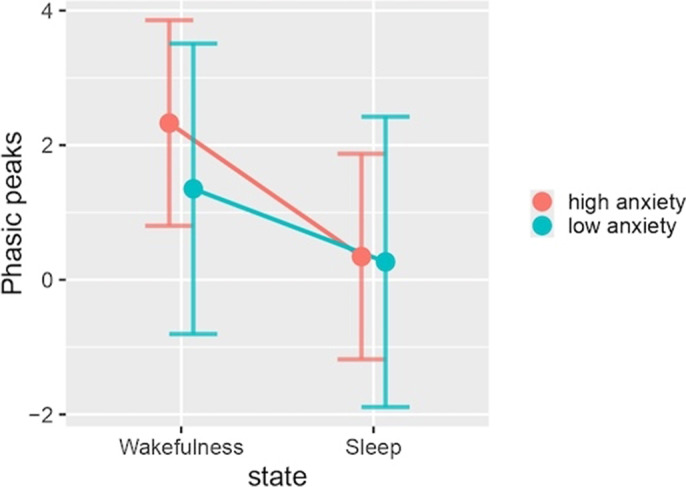

**Conclusions:**

These findings should be interpreted with caution due to the small sample size and the imbalance in the distribution of low (17%) versus high anxiety (83%) participants. Furthermore, anxiety is a multifaceted symptom that should be evaluated using a more comprehensive assessment tool. To validate these preliminary observations, it is essential to increase the sample size and use a more precise measure of anxiety.

**Disclosure of Interest:**

None Declared

